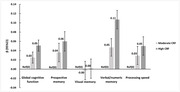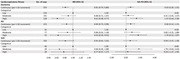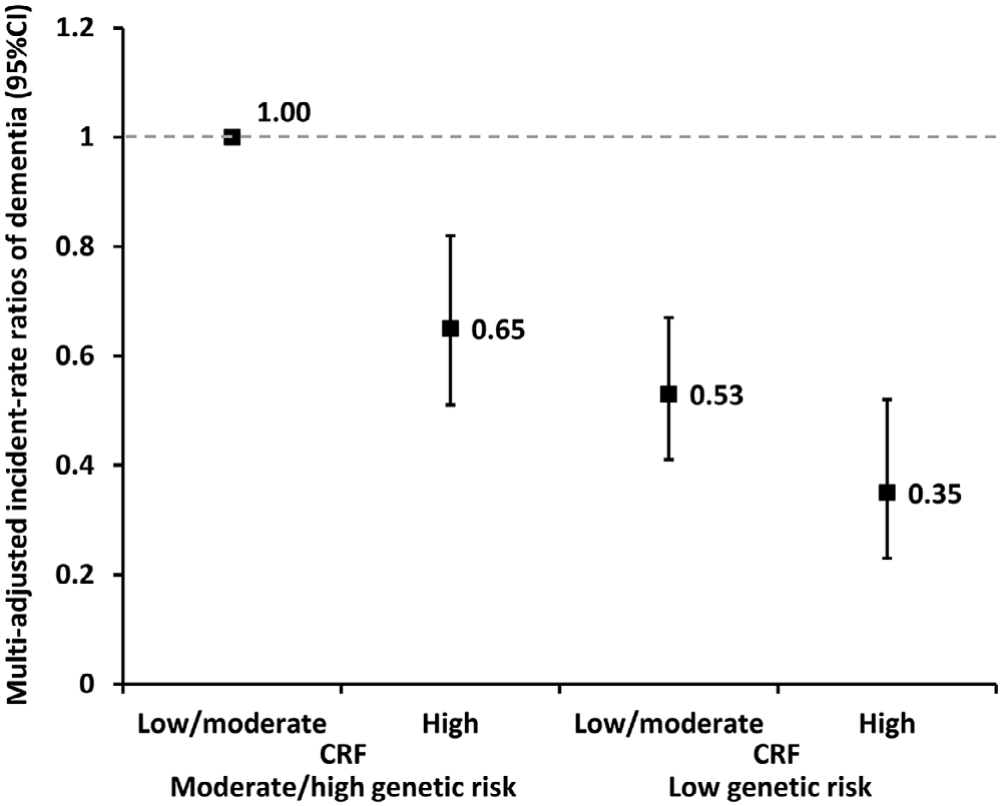# Association of cardiorespiratory fitness with dementia risk across different levels of genetic predisposition: a large community‐based longitudinal study

**DOI:** 10.1002/alz.084505

**Published:** 2025-01-09

**Authors:** Shuqi Wang, Liyao Xu, Wenzhe Yang, Jiao Wang, Abigail Dove, Xiuying Qi, Weili Xu

**Affiliations:** ^1^ School of Public Health, Tianjin Medical University, Tianjin China; ^2^ Tianjin Medical University, Tianjin China; ^3^ Department of Epidemiology, College of Preventive Medicine, the Army Medical University (Third Military Medical University), Chongqing China; ^4^ Aging Research Center, Karolinska Institutet, Stockholm Sweden; ^5^ School of Public Health, Tianjin Medical University, Tianjin, Tianjin China

## Abstract

**Background:**

Cardiorespiratory fitness (CRF), a proxy for cardiovascular and respiratory function, has been linked to health outcomes, but its association with cognition and dementia risk remains unclear. We aimed to investigate the association of CRF with domain‐specific cognitive function and dementia risk, taking genetic predisposition for dementia into account.

**Method:**

Within the UK Biobank, 61,214 dementia‐free participants aged 39‐70 (mean age: 56.33 ± 8.15, 51.96% were female) were followed for up to 16 years to detect incident dementia. CRF score (measured in metabolic equivalent of task [MET] units) was estimated using a 6‐min submaximal exercise test on a stationary bike and divided into tertiles (i.e., low, moderate, and high). Tests of global and domain‐specific cognitive functions (including prospective memory, visual memory, verbal/numeric memory, and processing speed) were administered at baseline. Dementia was identified based on medical history and medical records. Genetic predisposition for dementia was estimated using the polygenic risk score for Alzheimer’s disease (PRS_AD_), tertiled as low, moderate, or high level. Data were analyzed using linear regression, Poisson regression, and Laplace regression.

**Result:**

The age‐ and sex‐specific CRF ranged from ‐2.74 to 6.61 MET at baseline. In multi‐adjusted linear regression, compared to low CRF, high CRF was related to better global cognitive function (β [95% confidence interval]: 0.05 [0.04, 0.06]), prospective memory (0.06 [0.04, 0.08]), verbal/numeric memory (0.11 [0.09, 0.13]), and processing speed (0.05 [0.03, 0.07]). Over the follow‐up (median [interquartile range]: 11.52 [11.62–11.87] years), 553 individuals developed dementia. Compared to low CRF, the incidence rate ratio (IRR [95% CI]) of dementia was 0.61 (0.48–0.77) for high CRF, and the onset of dementia was delayed by 1.47 (95% CI: 0.64–2.30) years among people with high *vs*. low CRF. Among people with moderate/high genetic risk for dementia, high CRF can attenuate dementia risk by 35% (IRR = 0.65, 95% CI: 0.51–0.82). There was a trend of CRF to alleviate the genetic risk for dementia, but the additive interaction was not significant.

**Conclusion:**

High CRF is associated with better cognitive performance at baseline, and lower dementia risk over follow‐up. High CRF could mitigate the impact of genetic predisposition on dementia risk by almost 40%.